# Organizational structures of dialysis access care and the role of interventional nephrology: a Germany-wide survey

**DOI:** 10.1186/s12882-026-04988-w

**Published:** 2026-04-21

**Authors:** Martin Kächele, Lucas Bettac, Jens Dreyhaupt, Lena Schulte-Kemna, Bernd Schröppel

**Affiliations:** 1 Department of Internal Medicine I, Section of Nephrology, University Hospital Ulm, Albert-Einstein-Allee 23, 89081 Ulm, Germany; 2 Institute of Epidemiology and Medical Biometry, Ulm University, Schwabstrasse 13, 89075 Ulm, Germany

**Keywords:** Vascular access, Interventional nephrology, Dialysis access care, Dialysis, Health system reform

## Abstract

**Background:**

Ongoing changes in healthcare are expected to significantly reshape the hospital landscape in the coming years. Within this context and in order to respond appropriately, an up-to-date assessment of the structural and process quality of dialysis access care is essential.

**Methods:**

Between April and July 2024, 160 German inpatient nephrology departments were invited to participate in an anonymous online survey. Data were analysed descriptively.

**Results:**

A total of 64 hospitals responded (response rate: 40%). Of these, 23% (*n* = 15) were university hospitals, 45% (*n* = 29) were tertiary care providers, and 32% (*n* = 20) were primary (*n* = 1) or secondary care hospitals (*n* = 19). 19% (*n* = 12) were certified as a dialysis access centre. Availability of dialysis access surgery like arteriovenous fistulas (AVF), tunnelled-haemodialysis catheters (tHDC), and peritoneal dialysis (PD) catheters procedures was generally rated as good, with 80% performed in an inpatient setting. Satisfaction with access to AVF interventions trends to decrease with increasing hospital size. Overall, 58% of respondents favoured greater nephrology involvement, particularly in tHDC placement (92%), PD catheter placement (36%), and AVF procedures (44%). Nevertheless, only 25% reported planning to expand nephrological interventions. Key barriers included limited hands-on experience, lack of structured training curricula, inadequate infrastructure, and interdepartmental competition. Economic pressure and the shift toward outpatient care were cited as additional challenges.

**Conclusions:**

Most hospital nephrologists are satisfied with the service and timely availability of dialysis access surgery or interventions. While there is a strong desire to increase nephrological involvement, only a few hospitals plan to expand these services. Standardized training programs are needed to strengthen the field of interventional nephrology.

**Supplementary Information:**

The online version contains supplementary material available at 10.1186/s12882-026-04988-w.

## Background

Optimal dialysis access care requires interdisciplinary collaboration. To promote structured cooperation, a certification process for vascular access centres has been available in Germany for several years. Certification as an ‘Interdisciplinary Centre for Dialysis Access’, which is jointly supported by the national medical societies for nephrology, vascular surgery, angiology and interventional radiology, is implemented by a service organisation (ClarCert GmbH, Neu-Ulm, Germany). Centres can be certified as regional or reference centres. Provision of specialist departments and joint organisational structures at the site are required, as well as minimum annual numbers for dialysis access procedures (e.g. at least 50 surgical AVF interventions per year) [1]. The certification is voluntary and does not affect the authorisation to treat patients with dialysis access or reimbursement. Currently, only about one quarter of the approximately 160 hospitals with inpatient nephrology department are certified [2]. Outside of the certified centres, no quality data on dialysis access is collected or recorded in a register. Consequently, organizational and quality data for the majority of sites remain limited. Furthermore, there is limited knowledge regarding the responsibility for dialysis access within hospitals and the prevalence and role of interventional nephrology. Integrating interventional nephrology into dialysis access care could potentially enhance quality through faster, more specialized, and continuous patient support [3, 4]. Interventional nephrology has emerged as a global trend in both high-income and low-to-middle-income countries [5]. Germany is currently undergoing a structural hospital reform aiming at reorganising the healthcare landscape towards needs-based and quality-oriented care [6]: These shifts are likely to affect interdisciplinary organisation of dialysis access in hospitals. Consequently, it is therefore important to understand the degree of organisational framework, strengths and weaknesses and the evolving role of interventional nephrology within this new paradigm.

This survey aimed to (i) assess the current organization of dialysis access care across German inpatient nephrology departments, (ii) examine the role of interventional nephrology in this process, and (iii) identify barriers of broader involvement of nephrology in dialysis access creation.

## Methods

In April 2024, the heads of 160 nephrology departments in Germany were invited to participate in an anonymous online survey.

### Questionnaire

The 24-items questionnaire [Supplement: Questionnaire] was designed by the team of authors and underwent repeated internal review processes. The questionnaire had been tested by six internal and external physicians for functionality and ease of response before distribution. The questions focused on the structural organisation, process satisfaction and the role of interventional nephrology. The survey primarily utilized closed-ended questions, although open-questions relating to organisational structure allowed to capture less common constructs. A five-point Likert scale was used for questions regarding subjective assessment. Questions relating to the role of interventional nephrology and hospital reform allowed for free-text responses.

### Survey

The survey was conducted via the Unipark survey tool (Tivian GmbH, Cologne, Germany). The addresses of all hospitals with a nephrology department were identified to update existing lists. The requirement of ethics approval and informed consent was waived by the IRB, as our study utilized anonymized data. An online link to the survey was sent by email to department heads with a request to participate. The participation was voluntary and completing the survey implies consent. Duplicate submissions were prevented by restricting multiple entries from the same IP address. A reminder email was sent after two weeks. The survey was closed after three months.

### Statistical analysis

Surveys returned by July 2024 were analysed descriptively using Tivian EFS Reporting + and SPSS (SPSS IBM, Armonk, USA). Anonymous free-text responses were manually analysed for clusters of responses.

Continuous data were described by mean and standard deviation, categorical data as absolute frequencies and percentages. Associations between categorical variables were further investigated using Fisher’s exact test or chi-square test as appropriate. Furthermore, univariate proportional odds models were used to investigate the association of possible influencing variables (availability of vascular surgery or radiology, certification, structured cooperation) on satisfaction.

A two- sided p value of ≤ 0.05 was considered statistically significant. Due to the exploratory nature of this study, all results from statistical tests have to be interpreted as hypothesis-generating. An adjustment for multiple testing was not done. Statistical analysis was performed using, Microsoft Excel 365 (Microsoft, Redmond, WA, USA), IBM SPSS Statistics (Version 31; IBM Corp., Armonk, NY, USA) and SAS (version 9.4, The SAS Institute Cary, NC, USA).

## Results

### Hospital and physician characteristics

We were able to analyse total of 64 questionnaires, reflecting a response rate of 40%. Among the participating hospitals, 69% were tertiary care facilities (Level III), including 52% university hospitals (Level IIIU). Hospitals providing secondary care (Level II) accounted for 32%, while 2% were classified as primary care (Level I). For the analysis, Level I and II were summarized. The participating institutions reported an average of 10,855 dialysis sessions annually, with 3% performing fewer than 1,000 treatments per year (Table 1). There were 40 certified centres nationwide at the time of the survey. In our survey, 19% (*n* = 12) of the respondents reported to be a certified dialysis access centre. Nearly all of those (*n* = 10) were tertiary care facilities (Level III). Overall, 54 hospitals (84%) stated organized structures for cooperation having established on-site. Twelve hospitals (19%) indicated plans to apply for certification as interdisciplinary vascular access centre in the near future (Table 2).

**Table 1:** Organizational structure of the participating nephrology departments

	*N*	%
**Level of care**	64	
University hospital (Level IIIU)	15	23
Tertiary care hospital (Level III)	29	45
Secondary care hospital (Level II) + Primary care (Level I)	19 + 1	30 + 2
**Key figures** (mean ± SD)	64	
Number of beds Q1/ Q3 Minimum / Maximum	29 ± 15.3 20 / 40 0 / 70	
Dialysis treatments per year Q1/ Q3 Minimum / Maximum	10,855 ± 7,451 6000 / 12,000 10 / 46,500	
**Staffing** (mean ± SD)	64	
Chief physicians Q1 / Q3 Minimum / Maximum	1.1 ± 0.4 1 / 1 0 / 4	
Attending physicians Q1 / Q3 Minimum / Maximum	3.7 ± 3.5 2 / 4 0 / 23	
Fellows Q1 / Q3 Minimum / Maximum	7.3 ± 5.2 3 / 10 0 / 30	
**Further Nephrology training**	64	
Full further training	51	80
Partial training	10	16
No further training authorization	3	5

Definitions of hospital level: Level I: Basic care, Level II: Secondary care, at least two internal medicine and two surgical disciplines, intensive care, emergency medicine and at least three other disciplines. Level III: Tertiary care, 5 internal medicine and surgical disciplines, comprehensive emergency care, wide range of other specialties. Level IIIU: Same as Level III, University hospitals

Full training: Certification for specialist training in nephrology for 36 of the required 36 months. Partial: less than 36 months. No training: not accredited for specialty training

### Surgical and interventional expertise

Certification as a dialysis access centre requires a minimum of 50 primary AVF implantations and 30 AVF interventions per year. 13% had a vascular surgery department without expertise in AVF surgery, and 5% depended on cooperation with external surgical expertise. For key figures of hospital organization, compare (Table 2). Departments of radiology or vascular medicine with established experience in AVF interventions were available in 67% (*n* = 43). In isolated cases, AVF interventions were performed by vascular surgery and others. Endovascular AVF creation using techniques such as WavelinQ™ or Ellipsys™ was offered at 14 sites, predominantly by radiology or surgery, and in single cases by cardiology or nephrology.

**Table 2:** Hospitals: organizational structure of dialysis access service

	*n*	%
**Vascular surgery and expertise in AV fistula surgery**	64	100
Without expertise	8	13
With expertise	53	83
Not available at this location	0	0
Collaboration with other clinics/practices	3	5
**Interventional radiology/vascular medicine**	64	
No expertise	15	23
With expertise	43	67
Not available at the hospital	6	9
**Organizational structure (dialysis access centre)**	63	
Missing response	1	
No	9	15
Yes, structured cooperation	40	63
Certified dialysis access centre	12	19
Nephrological cooperation partner of a vascular access centre at another clinic	2	3
Certification planned	12	19
**Most procedures are performed on an** **outpatient / inpatient basis**	63	
Surgical procedures vascular access (initial implantation, revision)	9 / 54	14 / 86
Vascular access interventions	11 / 52	17 / 83

### Setting of procedures

The majority of centres performed these interventions predominantly in an inpatient setting. Surgical procedures accounted for 86% of cases, and 83% of AVF were also performed on inpatients.

### Availability of procedures

**Fig. 1:**
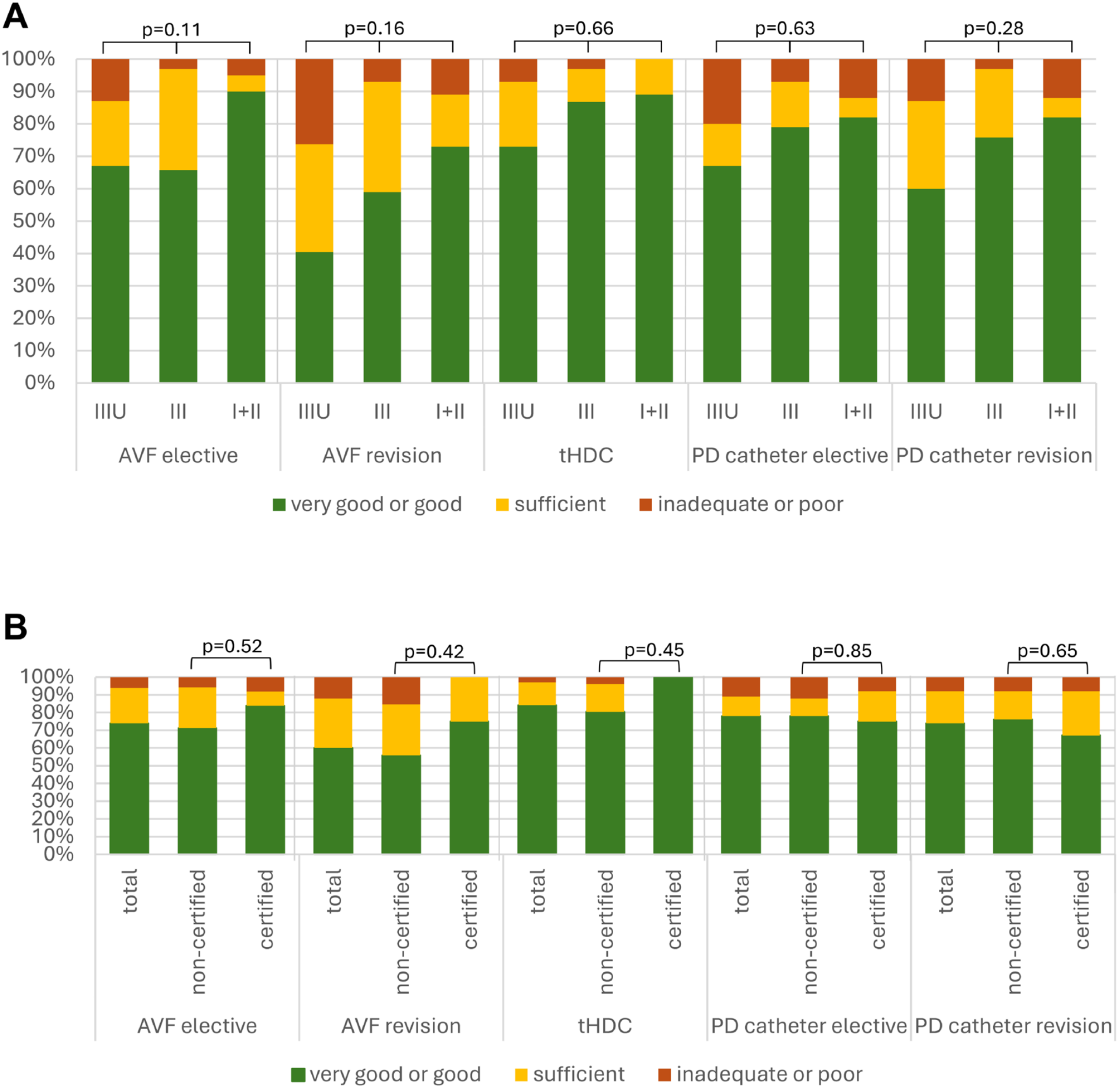
The availability of different types of procedures.(**A**) availability according to different levels of hospital care. Level IIIU: university hospitals; Level III: tertiary care, Level I + II: primary and secondary care. (**B**) availability in vascular access certified and non-certified hospitals.Respondents were asked about the availability of different types of procedures. The responses “good” and “very good” and “inadequate” and “poor” were combined for statistical analyses (Fisher’s Exact test for independency). Frequency of responses regarding satisfaction, expressed as a percentage. AVF: Arteriovenous fistula, tHDC: tunnelled haemodialysis catheter, PD: peritoneal dialysis; VA: vascular access

The timely availability of procedures was assessed on a five-point Likert scale ranging from “very good” to “poor” (Fig. 1). Elective AVF procedures were rated as very good or good by 74%, while 6% considered them inadequate or poor. AVF revisions were rated as very good or good by 60%, though 15% described availability as inadequate or poor. Tunnelled catheters received the most favourable assessment, with 85% rating their availability as very good or good and only 3% rating them inadequate. Elective PD catheter implantation and revision were rated positively by 78% and 74%, respectively, but 11% and 8% considered them inadequate or poor. No differences were found across the German federal states.

### Qualitative feedback

**Fig. 2:**
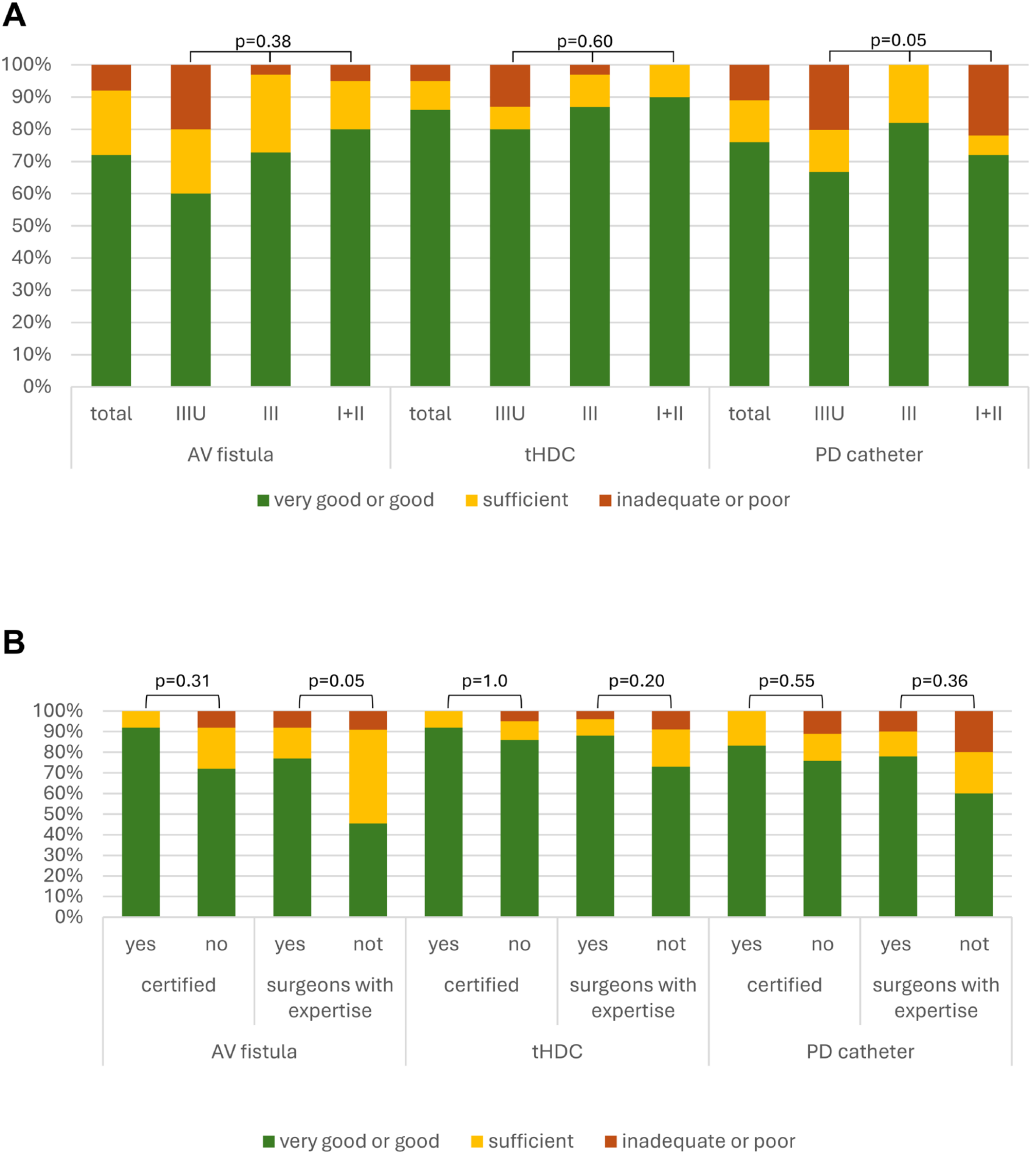
Satisfaction with care. (**A**) hospital level. Level IIIU: university hospitals; Level III: tertiary care, Level I + II: primary and secondary care. (**B**) organization level. Certified reflects accredited certification in vascular access. Respondents were asked about their satisfaction with various dialysis access options at the location. The responses “good” and “very good” as well as “poor” and “inadequate” were combined for statistical analyses (Fisher’s Exact test for independency). Frequency of responses regarding satisfaction, expressed as a percentage. *n* = 3 answers were missing for PD catheters (*n* = 61), all others *n* = 64. AVF: Arteriovenous fistula, tHDC: tunnelled haemodialysis catheter, PD: peritoneal dialysis; VA centre: vascular access centre

Assessment of the perceived quality for AV fistulas was positive, and rated as good or very good by 72%, with only 8% describing it as inadequate. For tunnelled catheters, the proportion satisfied with care quality was 86%, while 5% expressed dissatisfaction. PD catheters were rated good or very good by 76%, 11% reported dissatisfaction (Fig. 2). To identify possible associations with satisfaction, influencing variables like availability of vascular surgery or radiology, accredited certification or structured cooperation were investigated using a univariate proportional odds model. No statistically significant influencing variables were found (for details compare Supplement Table S1).

### Availability of interventional nephrology

Many nephrology departments reported to be already engaged in interventional procedures. The majority reported performing kidney biopsies (95%), placing non-tunnelled dialysis catheters (92%) and tHDC (67%). 44% reported to perform peritoneal dialysis (PD) catheters procedures [Supplement Fig. S1]. The high response frequency contradicts the answers to the following, more detailed questions for AVFs and interventions: While 28% indicated they performed endovascular AVF placement, 36% reported surgical AVF surgery, and 38% AVF interventions, later only 5% confirmed any nephrological involvement in surgical AVF creation. Asking in detail on nephrology’s involvement in AVF procedures (participative or autonomous), 11% reported performing AVF interventions and 6% stated to create endovascular AV fistulas. Due to discrepancies in the responses to questions that overlap in content regarding nephrologists’ activities, no further analysis was carried out based on these answers.

### Interventional nephrology and barriers

58% considered nephrology involvement in vascular access procedures important as a complementary service. The proportion in favour of nephrology participation was highest for tunnelled catheters (92%, *n* = 36)), followed by AVF (44%, *n* = 17), PD catheters (36%, *n* = 14), and AVF interventions (28%, *n* = 11). Despite this, 75% (*n* = 48) of departments stated that they did not plan to expand their interventional spectrum. Where an expansion was reported, this primarily involved interventional AVFs (13%, *n* = 8) and PD catheters (11%, *n* = 7).

**Fig. 3:**
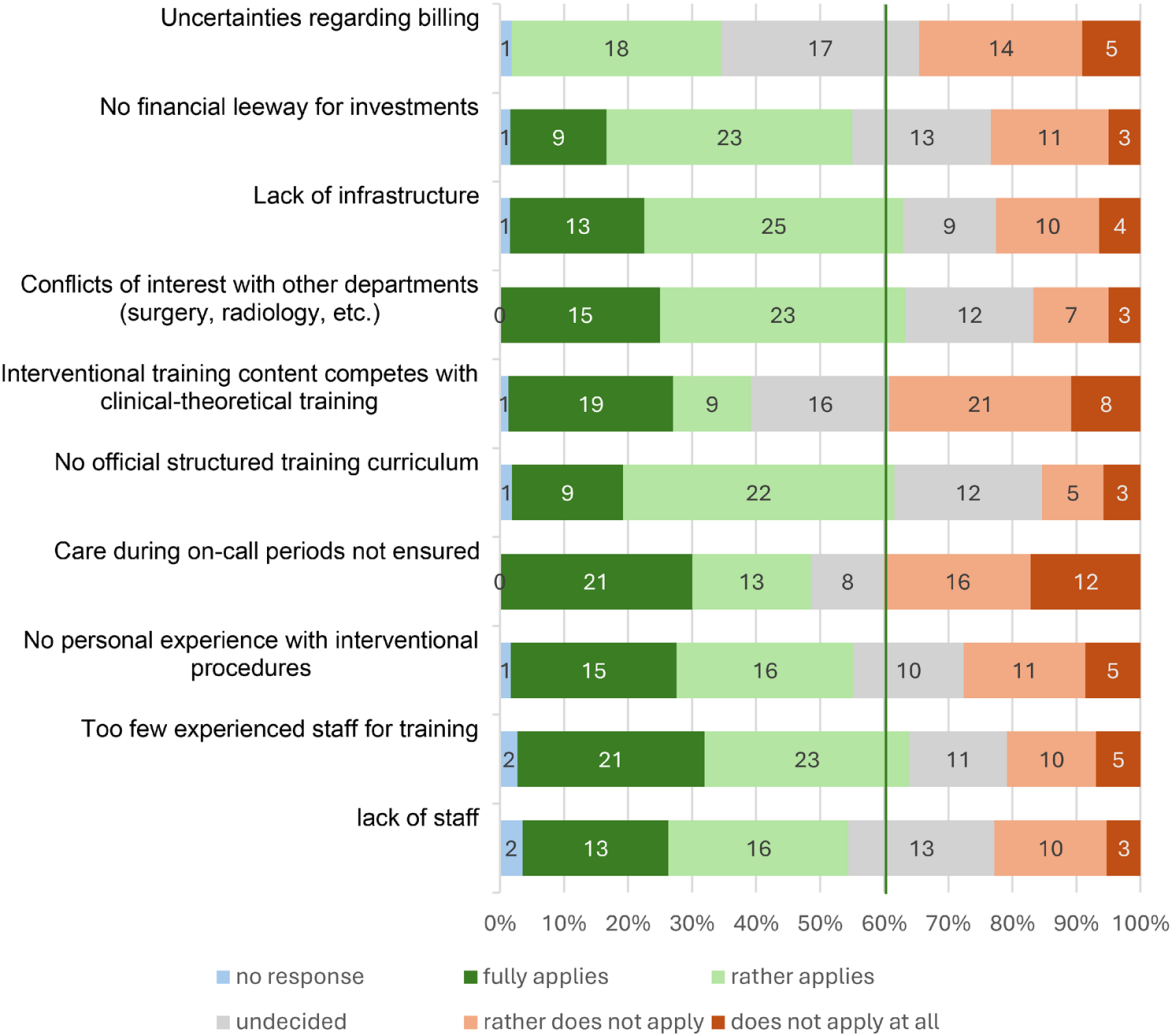
Barriers for interventional nephrology. Potential barriers to interventional nephrology using a 5-point Likert scale matrix. Response options ranged from 1: absolutely true to 5: not true at all. The numbers correspond to the frequency of responses (n). For better visualisation of the four items with the highest approval ratings, a line was added at 60% approval

Respondents identified several obstacles to the establishment or expansion of interventional nephrology programs (Fig. 3) The most frequently reported barriers included the absence of structured training curricula, lack of personal procedural experience, insufficient infrastructure, and conflicts of interest with other departments, particularly surgery. In addition, the ongoing trend toward outpatient care and increasing financial pressure were perceived as major challenges. Only a minority of the participants in the survey made use of the free-text option, without contributing additional aspects.

## Discussion

The primary aim of our survey was to characterize the current organization and status of dialysis access programs in Germany. Among the 160 nephrology departments with at least two nephrologists, we achieved a response rate of 40%.

Three main findings emerge from our analysis: first, most hospitals have structured their interdisciplinary collaboration for dialysis access and are satisfied with the current situation, particularly in larger non-university hospitals; second, dialysis access procedures are predominantly performed in an inpatient setting; and third, a majority of respondents emphasized the importance of expanding involvement of nephrology in the placement of peritoneal dialysis (PD) and haemodialysis catheters.

Overall, vascular access care structures continue to be broadly interdisciplinary and appear well organized. The majority of respondents reported satisfaction with the timeliness and quality of care. However, closer examination reveals structural differences that highlight areas of deficit. University hospitals, in particular, reported more frequently perceived challenges in ensuring timely availability of access procedures (when looking at the proportion rated as “very good-good”) across all categories. By contrast, tertiary, secondary, and primary care hospitals appeared better organized to perform time-sensitive interventions. This observation raises important questions regarding the efficiency of care coordination in university hospitals and indicates a need for targeted process and structural adjustments. PD catheters are an exception, as patient satisfaction appears to be highest in tertiary care hospitals. One possible explanation for this could be competing interests at university hospitals and a shortage of general surgeons with experience at smaller hospitals. In university hospitals in particular, dialysis access procedures compete with major surgical procedures, which has a negative impact on planning and prioritisation from the surgeon’s perspective. Outsourcing and thus relieving the burden on surgical units might be helpful, e.g. through nephrology-led procedure rooms.

The survey also shows that satisfaction with care is considerably higher within certified dialysis access centres (not statistically significant), though the perceived satisfaction can be explained, at least in part, by good availability. This suggests that structured, binding agreements within interdisciplinary care pathways are effective in ensuring quality and timeliness. At the same time, the lower satisfaction at sites without surgical AVF expertise underscores the necessity of establishing clear minimum standards and consistent on-site expertise.

Deficits in the timely availability of revision procedures for both AV fistulas and PD catheters were reported, even at certified centres. Therefore, particular concern has to be noted with respect to PD catheters. Compared with other forms of dialysis access, service for PD catheters were rated notably lower. The absence of numeric performance thresholds for PD catheters in the current certification framework suggests that essential aspects of real-world care delivery remain insufficiently addressed. Expanding certification and quality criteria to include specific PD-related measures could therefore be important to ensure comprehensive and needs-based access care.

The planned health care reform aims to improve quality by concentrating specialised services [6]. In contrast to other European countries and worldwide practice, outpatient dialysis access care in Germany remains limited, with most patients treated in hospitals. Model calculations indicate that the financing cap introduced by the reform could affect funding for approximately 40% of low-volume vascular surgery departments [7]. Our data reveal a non-significant trend between university and non-university hospitals in the timely provision of dialysis access, which maybe could further aggravate by these changes [8]. Strengthening interventional nephrology might be a strategy, which could align regional demand with service provision and ensure that all necessary structures are available [9].

Interventional nephrology plays a minor role in many European countries with comparable healthcare systems [10], with expertise concentrated in only a few centres. In some countries, such as Slovenia and Italy, interventional nephrology has developed and contributes significantly to nationwide care [11]. A publication from the UK shows a benefit for care after the program was rolled out [12].

In our survey, a majority considered nephrology involvement in dialysis access procedures important as a complementary service, but most did not consider to expand the interventional spectrum, reasons for this were multifactorial. They included limited personnel and time resources, insufficient procedural expertise within nephrology, lack of operating room capacity and technical infrastructure, and the perceived costs and time involved in establishing new service structures. In many hospitals, close collaborations with vascular surgery or radiology were already well established, and respondents often considered these collaborations sufficient to cover local needs. Persistent challenges also included staff shortages and, importantly, the absence of an official training curriculum in interventional nephrology.

Recent publications in Europe have set out best-practice recommendations for various dialysis access methods for nephrologists [13, 14]. The recently launched N-PATH consortium aims to develop structured training programs for young nephrologists, with a strong emphasis on procedural and interventional skills [15]. Similar efforts have been advanced by the American Society of Diagnostic and Interventional Nephrology (ASDIN), which has introduced structured training pathways and certifications to promote standardization and quality assurance [16, 17]. Our survey indicates that some German hospitals have the necessary structures to initiate such training programmes.

### Limitations

Our study has several limitations. There is a bias towards larger tertiary care hospitals. Primary and secondary hospitals with relatively smaller nephrology departments are underrepresented. In Germany there are no nationwide databases or statistics on hospital structure available, therefore our data are based solely on self-reported information. Ambiguities in responses regarding independent nephrological activity hinder definite conclusions about current practice. The conflicting responses to survey items suggest that the actual penetration of interventional nephrology in practice may be lower than our findings indicate.

Our study was conducted in Germany and the results cannot be readily extrapolated to other countries. For example, unlike in many other countries, in Germany dialysis access is established in an inpatient setting. The reasons for this are manifold and include rigid sector separation and low reimbursement rates posing major obstacles [18].

The functionality of dialysis access care is the result of a delicate interplay between available structural resources, financial funding, and the focus of the national dialysis program (e.g. peritoneal versus hemodialysis program), which can lead to significantly different outcomes even within same world regions. Consequently, our data reflect a specific national health care context. Yet, highly developed healthcare systems are affected by similar changes that have been observed in Germany. Resource allocation challenges often compromise technically less complex procedures like dialysis access. Our findings therefore may inform international efforts to optimize nephrology service organization and delivery [19]. Interventional Nephrology, as a single-source model of care, meets these needs by combining first-hand knowledge of dialysis patients’ requirements with technically simplified approaches, such as ultrasound guidance [20, 21]. This enables timely, appropriate, and effective interventions. The ability to manage dialysis accesses strengthens fellows’ awareness and competence in this often-neglected subject.

## Conclusion

Dialysis access care in Germany is well-structured and primarily inpatient-based, with high center satisfaction. Interventional nephrology is currently mainly limited to catheter procedures. However, progress is hindered by training, infrastructure, and interdepartmental dynamics, rather than lack of perceived importance. This survey focuses on organizational structures and perceptions, excluding patient outcomes or costs.

## Supplementary Information

Below is the link to the electronic supplementary material.

Supplementary Material 1

Supplementary Material 2

## Data Availability

The dataset supporting the conclusions of this article is available from the corresponding author on reasonable request.

## Abbreviations

AVF: Arterio-venous fistula
PD: Peritoneal dialysis
SD: Standard deviation
tHDC: Tunnelled haemodialysis catheter
VA: Vascular access
