# Supplementary material for: Organizational structures of dialysis access care and the role of interventional nephrology: a Germany-wide survey
Source: BMC Nephrol. 2026 Apr 21;27:254. doi: 10.1186/s12882-026-04988-w (PMC13104340; doi:10.1186/s12882-026-04988-w)
Supplement: Supplementary file 1 — Supplementary Material 1 [file 12882_2026_4988_MOESM1_ESM.docx]

**Supplement Figure 1: Availability of procedures by state**

|  | | **AV fistula elective** | | | | | | | | total | |
| --- | --- | --- | --- | --- | --- | --- | --- | --- | --- | --- | --- |
|  |  | very good | | good | | sufficient | | inadequate | |  |  |
|  |  | N | % | N | % | N | % | N | % | N | % |
| state | Baden-Württemberg | 3 | 14% | 2 | 8% | 2 | 15% | 1 | 25% | 8 | 13% |
|  | Bavaria | 3 | 14% | 3 | 12% | 1 | 8% | 0 | 0% | 7 | 11% |
|  | Rhineland-Palatinate | 0 | 0% | 1 | 4% | 0 | 0% | 0 | 0% | 1 | 2% |
|  | Saarland | 0 | 0% | 1 | 4% | 0 | 0% | 0 | 0% | 1 | 2% |
|  | Hesse | 2 | 10% | 3 | 12% | 0 | 0% | 1 | 25% | 6 | 9% |
|  | Thuringia | 0 | 0% | 1 | 4% | 1 | 8% | 0 | 0% | 2 | 3% |
|  | North Rhine-Westphalia | 7 | 33% | 5 | 19% | 1 | 8% | 1 | 25% | 14 | 22% |
|  | Saxony | 1 | 5% | 1 | 4% | 4 | 31% | 0 | 0% | 6 | 9% |
|  | Saxony-Anhalt | 1 | 5% | 1 | 4% | 1 | 8% | 0 | 0% | 3 | 5% |
|  | Lower Saxony | 0 | 0% | 3 | 12% | 0 | 0% | 0 | 0% | 3 | 5% |
|  | Mecklenburg-Vorpommern | 1 | 5% | 0 | 0% | 1 | 8% | 0 | 0% | 2 | 3% |
|  | Brandenburg | 2 | 10% | 1 | 4% | 0 | 0% | 0 | 0% | 3 | 5% |
|  | Bremen | 0 | 0% | 1 | 4% | 0 | 0% | 0 | 0% | 1 | 2% |
|  | Berlin | 1 | 5% | 2 | 8% | 1 | 8% | 0 | 0% | 4 | 6% |
|  | Schleswig-Hostein | 0 | 0% | 0 | 0% | 0 | 0% | 1 | 25% | 1 | 2% |
|  | Hamburg | 0 | 0% | 1 | 4% | 1 | 8% | 0 | 0% | 2 | 3% |
| total | | 21 | 100% | 26 | 100% | 13 | 100% | 4 | 100% | 64 | 100% |

Pearson-Chi-Square 46,856, p=0,396

|  | | **AV fistula revision** | | | | | | | | | | total | |
| --- | --- | --- | --- | --- | --- | --- | --- | --- | --- | --- | --- | --- | --- |
|  |  | very good | | good | | sufficient | | inadequate | | poor | |  |  |
|  |  | N | % | N | % | N | % | N | % | N | % | N | % |
| state | Baden-Württemberg | 1 | 5% | 2 | 11% | 1 | 6% | 2 | 33% | 2 | 100% | 8 | 12,5% |
|  | Bavaria | 1 | 5% | 4 | 21% | 2 | 11% | 0 | 0% | 0 | 0% | 7 | 10,9% |
|  | Rhineland-Palatinate | 0 | 0% | 0 | 0% | 0 | 0% | 1 | 17% | 0 | 0% | 1 | 1,6% |
|  | Saarland | 0 | 0% | 1 | 5% | 0 | 0% | 0 | 0% | 0 | 0% | 1 | 1,6% |
|  | Hesse | 4 | 21% | 1 | 5% | 1 | 6% | 0 | 0% | 0 | 0% | 6 | 9,4% |
|  | Thuringia | 1 | 5% | 0 | 0% | 1 | 6% | 0 | 0% | 0 | 0% | 2 | 3,1% |
|  | North Rhine-Westphalia | 6 | 32% | 3 | 16% | 4 | 22% | 1 | 17% | 0 | 0% | 14 | 21,9% |
|  | Saxony | 1 | 5% | 1 | 5% | 3 | 17% | 1 | 17% | 0 | 0% | 6 | 9,4% |
|  | Saxony-Anhalt | 0 | 0% | 2 | 11% | 1 | 6% | 0 | 0% | 0 | 0% | 3 | 4,7% |
|  | Lower Saxony | 3 | 16% | 0 | 0% | 0 | 0% | 0 | 0% | 0 | 0% | 3 | 4,7% |
|  | Mecklenburg-Vorpommern | 0 | 0% | 1 | 5% | 1 | 6% | 0 | 0% | 0 | 0% | 2 | 3,1% |
|  | Brandenburg | 1 | 5% | 1 | 5% | 1 | 6% | 0 | 0% | 0 | 0% | 3 | 4,7% |
|  | Bremen | 0 | 0% | 0 | 0% | 1 | 6% | 0 | 0% | 0 | 0% | 1 | 1,6% |
|  | Berlin | 1 | 5% | 2 | 11% | 0 | 0% | 1 | 17% | 0 | 0% | 4 | 6,3% |
|  | Schleswig-Hostein | 0 | 0% | 0 | 0% | 1 | 6% | 0 | 0% | 0 | 0% | 1 | 1,6% |
|  | Hamburg | 0 | 0% | 1 | 5% | 1 | 6% | 0 | 0% | 0 | 0% | 2 | 3,1% |
| total | | 19 | 100% | 19 | 100% | 18 | 100% | 6 | 100% | 2 | 100% | 64 | 100,0% |

Pearson-Chi-square 61,740, p=0,414

|  | | **tunneled hemodialysis catheter** | | | | | | | | | | total | |
| --- | --- | --- | --- | --- | --- | --- | --- | --- | --- | --- | --- | --- | --- |
|  |  | missing | | very good | | good | | sufficient | | inadequate | |  |  |
|  |  | N | % | N | % | N | % | N | % | N | % | N | % |
| state | Baden-Württemberg | 1 | 100% | 2 | 6% | 3 | 17% | 1 | 13% | 1 | 50% | 8 | 13% |
|  | Bavaria | 0 | 0% | 4 | 11% | 3 | 17% | 0 | 0% | 0 | 0% | 7 | 11% |
|  | Rhineland-Palatinate | 0 | 0% | 1 | 3% | 0 | 0% | 0 | 0% | 0 | 0% | 1 | 2% |
|  | Saarland | 0 | 0% | 1 | 3% | 0 | 0% | 0 | 0% | 0 | 0% | 1 | 2% |
|  | Hesse | 0 | 0% | 5 | 14% | 1 | 6% | 0 | 0% | 0 | 0% | 6 | 9% |
|  | Thuringia | 0 | 0% | 1 | 3% | 1 | 6% | 0 | 0% | 0 | 0% | 2 | 3% |
|  | North Rhine-Westphalia | 0 | 0% | 9 | 26% | 3 | 17% | 2 | 25% | 0 | 0% | 14 | 22% |
|  | Saxony | 0 | 0% | 1 | 3% | 1 | 6% | 3 | 38% | 1 | 50% | 6 | 9% |
|  | Saxony-Anhalt | 0 | 0% | 2 | 6% | 0 | 0% | 1 | 13% | 0 | 0% | 3 | 5% |
|  | Lower Saxony | 0 | 0% | 3 | 9% | 0 | 0% | 0 | 0% | 0 | 0% | 3 | 5% |
|  | Mecklenburg-Vorpommern | 0 | 0% | 1 | 3% | 1 | 6% | 0 | 0% | 0 | 0% | 2 | 3% |
|  | Brandenburg | 0 | 0% | 1 | 3% | 1 | 6% | 1 | 13% | 0 | 0% | 3 | 5% |
|  | Bremen | 0 | 0% | 0 | 0% | 1 | 6% | 0 | 0% | 0 | 0% | 1 | 2% |
|  | Berlin | 0 | 0% | 3 | 9% | 1 | 6% | 0 | 0% | 0 | 0% | 4 | 6% |
|  | Schleswig-Hostein | 0 | 0% | 1 | 3% | 0 | 0% | 0 | 0% | 0 | 0% | 1 | 2% |
|  | Hamburg | 0 | 0% | 0 | 0% | 2 | 11% | 0 | 0% | 0 | 0% | 2 | 3% |
| total | | 1 | 100% | 35 | 100% | 18 | 100% | 8 | 100% | 2 | 100% | 64 | 100% |

Pearson-Chi-Square 45,850, p=0,911

|  | | **PD catheter elective** | | | | | | | | | | | | total | |
| --- | --- | --- | --- | --- | --- | --- | --- | --- | --- | --- | --- | --- | --- | --- | --- |
|  |  | missi+C165:N165ng | | very good | | good | | sufficient | | inadequate | | poor | |  |  |
|  |  | N | % | N | % | N | % | N | % | N | % | N | % | N | % |
| state | Baden-Württemberg | 0 | 0% | 1 | 5% | 3 | 11% | 2 | 29% | 0 | 0% | 2 | 66,7% | 8 | 12,5% |
|  | Bavaria | 0 | 0% | 3 | 14% | 3 | 11% | 0 | 0% | 0 | 0% | 1 | 33,3% | 7 | 10,9% |
|  | Rhineland-Palatinate | 0 | 0% | 0 | 0% | 1 | 4% | 0 | 0% | 0 | 0% | 0 | 0,0% | 1 | 1,6% |
|  | Saarland | 0 | 0% | 0 | 0% | 1 | 4% | 0 | 0% | 0 | 0% | 0 | 0,0% | 1 | 1,6% |
|  | Hesse | 0 | 0% | 4 | 19% | 2 | 7% | 0 | 0% | 0 | 0% | 0 | 0,0% | 6 | 9,4% |
|  | Thuringia | 0 | 0% | 1 | 5% | 1 | 4% | 0 | 0% | 0 | 0% | 0 | 0,0% | 2 | 3,1% |
|  | North Rhine-Westphalia | 2 | 100% | 8 | 38% | 2 | 7% | 1 | 14% | 1 | 25% | 0 | 0,0% | 14 | 21,9% |
|  | Saxony | 0 | 0% | 0 | 0% | 4 | 15% | 2 | 29% | 0 | 0% | 0 | 0,0% | 6 | 9,4% |
|  | Saxony-Anhalt | 0 | 0% | 1 | 5% | 1 | 4% | 0 | 0% | 1 | 25% | 0 | 0,0% | 3 | 4,7% |
|  | Lower Saxony | 0 | 0% | 0 | 0% | 3 | 11% | 0 | 0% | 0 | 0% | 0 | 0,0% | 3 | 4,7% |
|  | Mecklenburg-Vorpommern | 0 | 0% | 0 | 0% | 2 | 7% | 0 | 0% | 0 | 0% | 0 | 0,0% | 2 | 3,1% |
|  | Brandenburg | 0 | 0% | 1 | 5% | 0 | 0% | 1 | 14% | 1 | 25% | 0 | 0,0% | 3 | 4,7% |
|  | Bremen | 0 | 0% | 0 | 0% | 1 | 4% | 0 | 0% | 0 | 0% | 0 | 0,0% | 1 | 1,6% |
|  | Berlin | 0 | 0% | 1 | 5% | 2 | 7% | 1 | 14% | 0 | 0% | 0 | 0,0% | 4 | 6,3% |
|  | Schleswig-Hostein | 0 | 0% | 0 | 0% | 0 | 0% | 0 | 0% | 1 | 25% | 0 | 0,0% | 1 | 1,6% |
|  | Hamburg | 0 | 0% | 1 | 5% | 1 | 4% | 0 | 0% | 0 | 0% | 0 | 0,0% | 2 | 3,1% |
| total | | 2 | 100% | 21 | 100% | 27 | 100% | 7 | 100% | 4 | 100% | 3 | 100,0% | 64 | 100,0% |

Pearson-Chi-Square 74,306, p=0,501

|  | | **PD catheter revision** | | | | | | | | | | | | total | |
| --- | --- | --- | --- | --- | --- | --- | --- | --- | --- | --- | --- | --- | --- | --- | --- |
|  |  | missing | | very good | | good | | sufficient | | inadequate | | poor | |  |  |
|  |  | N | % | N | % | N | % | N | % | N | % | N | % | N | % |
| state | Baden-Württemberg | 0 | 0% | 1 | 4% | 3 | 13% | 3 | 27% | 0 | 0% | 1 | 50,0% | 8 | 13% |
|  | Bavaria | 0 | 0% | 4 | 17% | 2 | 9% | 0 | 0% | 0 | 0% | 1 | 50,0% | 7 | 11% |
|  | Rhineland-Palatinate | 0 | 0% | 0 | 0% | 0 | 0% | 0 | 0% | 1 | 33% | 0 | 0,0% | 1 | 2% |
|  | Saarland | 0 | 0% | 0 | 0% | 1 | 4% | 0 | 0% | 0 | 0% | 0 | 0,0% | 1 | 2% |
|  | Hesse | 0 | 0% | 4 | 17% | 2 | 9% | 0 | 0% | 0 | 0% | 0 | 0,0% | 6 | 9% |
|  | Thuringia | 0 | 0% | 1 | 4% | 0 | 0% | 1 | 9% | 0 | 0% | 0 | 0,0% | 2 | 3% |
|  | North Rhine-Westphalia | 2 | 100% | 6 | 26% | 4 | 17% | 1 | 9% | 1 | 33% | 0 | 0,0% | 14 | 22% |
|  | Saxony | 0 | 0% | 0 | 0% | 4 | 17% | 2 | 18% | 0 | 0% | 0 | 0,0% | 6 | 9% |
|  | Saxony-Anhalt | 0 | 0% | 1 | 4% | 2 | 9% | 0 | 0% | 0 | 0% | 0 | 0,0% | 3 | 5% |
|  | Lower Saxony | 0 | 0% | 2 | 9% | 1 | 4% | 0 | 0% | 0 | 0% | 0 | 0,0% | 3 | 5% |
|  | Mecklenburg-Vorpommern | 0 | 0% | 0 | 0% | 1 | 4% | 1 | 9% | 0 | 0% | 0 | 0,0% | 2 | 3% |
|  | Brandenburg | 0 | 0% | 1 | 4% | 0 | 0% | 2 | 18% | 0 | 0% | 0 | 0,0% | 3 | 5% |
|  | Bremen | 0 | 0% | 0 | 0% | 1 | 4% | 0 | 0% | 0 | 0% | 0 | 0,0% | 1 | 2% |
|  | Berlin | 0 | 0% | 2 | 9% | 1 | 4% | 1 | 9% | 0 | 0% | 0 | 0,0% | 4 | 6% |
|  | Schleswig-Hostein | 0 | 0% | 0 | 0% | 0 | 0% | 0 | 0% | 1 | 33% | 0 | 0,0% | 1 | 2% |
|  | Hamburg | 0 | 0% | 1 | 4% | 1 | 4% | 0 | 0% | 0 | 0% | 0 | 0,0% | 2 | 3% |
| total | | 2 | 100% | 23 | 100% | 23 | 100% | 11 | 100% | 3 | 100% | 2 | 100,0% | 64 | 100% |

Pearson-Chi-Square 87,046, p=0,161
