# Supplementary material for: Organizational structures of dialysis access care and the role of interventional nephrology: a Germany-wide survey
Source: BMC Nephrol. 2026 Apr 21;27:254. doi: 10.1186/s12882-026-04988-w (PMC13104340; doi:10.1186/s12882-026-04988-w)
Supplement: Supplementary file 2 — Supplementary Material 2 [file 12882_2026_4988_MOESM2_ESM.docx]

**Supplements**

**Questionnaire (translated from German)**

1. My department is part of a:

• University hospital (Level IIIU)

• Tertiary care hospital (Level III)

• Secondary care hospital with extended spectrum (at least 3 internal medicine service groups, Level II)

• Primary care hospital (Level I)

2. In which federal state is your hospital located?

• Baden-Württemberg

• Bavaria

• Berlin

• Brandenburg

• Bremen

• Hamburg

• Hesse

• Mecklenburg-Vorpommern

• Lower Saxony

• North Rhine-Westphalia

• Rhineland-Palatinate

• Saarland

• Saxony

• Saxony-Anhalt

• Schleswig-Holstein

• Thuringia

3. How many beds does your nephrology department have?

4. Approximately how many dialysis treatments (inpatient + outpatient) are performed per year in your nephrology department/clinic?

5. What is your staff structure in nephrology (chief physicians – senior physicians – residents)?

• Head of Department / Chief Physician

• Senior Physician

• Resident Physicians

6. Scope of training authorization for nephrology specialization at your site:

• Full training

• Partial training

• No training

7. Vascular surgery department at your hospital?

• Yes, but without expertise in vascular access surgery

• Yes, with expertise in vascular access surgery

• No, no cooperation with external vascular surgery

• No, cooperation with external vascular surgery (hospital/outpatient clinic)

8. Procedures on vascular access (initial creation and revisions) are currently performed predominantly:

• Outpatient

• Inpatient

9. Department of interventional radiology / angiology at your hospital?

• Yes, but without expertise in vascular access interventions

• Yes, with expertise in vascular access interventions

• No (continue on next page)

If no:

10. Vascular access interventions (PTA, etc.) are predominantly carried out by:

• No option / collaboration available

• External collaboration partner (practice/clinic)

• Surgery (in-house)

• Nephrology (in-house)

• Cardiology (in-house)

• Angiology (in-house)

• Other hospital department: ______

11. Interventions on vascular access are currently predominantly performed:

• Outpatient

• Inpatient

12. Interventional vascular access creation (e.g. Ellipsys, WavelinQ) at

• This hospital location

• Not offered

• Offered (external to location)

If “This hospital location”:

13. Interventional vascular access creation (e.g. Ellipsys, WavelinQ) at this location is performed by:

• Radiology

• Surgery

• Nephrology

• Cardiology

• Other: ______

14. Is there an organized structure for creating dialysis access (vascular access centre) at your location? (multiple selection):

• No

• Yes, structured collaboration with vascular surgery and interventional radiology/angiology

• Yes, hospital is a certified regional vascular access center

• Yes, hospital is a certified vascular access reference center

• We are a nephrology cooperation partner of a vascular access center at another hospital

• We are planning certification as a vascular access center or reference center

15. Which procedures are performed independently by nephrology at your location?

• Kidney biopsy

• Non-tunnelled dialysis catheters (Shaldon, etc.)

• Tunnelled central venous catheters

• Peritoneal dialysis catheters

• Interventional vascular access creation (Ellipsys, WavelinQ)

• Surgical vascular access creation

• Interventional vascular access procedures (PTA, etc.)

16. Nephrology involvement in surgical vascular access procedures:

• No involvement

• Operations performed cooperatively with other departments (nephrology actively participates in surgery)

• Nephrology independently performs vascular access surgeries (non-interventional procedures)

17. Nephrology involvement in interventional vascular access procedures:

• No involvement

• Nephrology performs interventions (e.g. PTA) independently

• Interventions performed cooperatively with other departments (nephrology actively participates in interventions)

18. The timeliness of dialysis access care for nephrology patients at my location (current state) I consider to be:

1: Very good | 2: Good | 3: Sufficient | 4: Inadequate | 5: Poor

• Elective vascular access creation

• Vascular access revision

• Tunnelled central venous catheter placement

• Elective peritoneal dialysis catheter placement

• Peritoneal dialysis catheter revision

19. Satisfaction with dialysis access care for nephrology patients at my location (current state), regarding:

1: Very good | 2: Good | 3: Sufficient | 4: Inadequate | 5: Poor

• AV vascular access (fistula/graft)

• Tunnelled central venous catheter placement

• Peritoneal dialysis catheter

20. Do you plan to perform procedures in the future that have not been established in your nephrology department so far?

• No / rather not

• Yes

20.1 If yes:

• Tunnelled central venous catheters

• Peritoneal dialysis catheters

• Interventional vascular access (Ellipsys, WavelinQ)

• Surgical vascular access procedures

• Vascular access interventions

• Other: ______

• Don’t know yet

21. As a complementary service to improve existing structures, I consider it important that nephrology itself performs certain procedures:

• No

• Yes, in the following cases

21.1 If yes:

• Tunnelled central venous catheters

• Peritoneal dialysis catheters

• Interventional vascular access creation (Ellipsys, WavelinQ)

• Surgical vascular access creation

• Vascular access interventions

21.2 Possible main reason for assessment of nephrology’s (non-)involvement: ______

22. How do you assess the impact of the planned hospital structure reform on dialysis access provision at your location?

• Improvement

• No change expected

• Deterioration

• No statement / no assessment possible

22.1 Optional comment: ______

23. Which of the following reasons do you see at your location as obstacles to establishing or continuing a program for “interventional nephrology”?

(fully applies – rather applies – undecided – rather does not apply – does not apply at all)

• Lack of staff

• Too few experienced staff for training

• No personal experience with interventional procedures

• Care during on-call periods not ensured

• No official structured training curriculum

• Interventional training content competes with clinical-theoretical training

• Conflicts of interest with other departments (surgery, radiology, etc.)

• Lack of infrastructure

• No financial leeway for investments

• Uncertainties regarding billing

23.1 Other (free text, opinion or comment): ______

**Figure S1 Procedures performed within the nephrology department**

Frequency of responses to the question 15: "Which procedures are performed by nephrology itself?" In separate questions for the way of involvement in “interventional vascular access procedures”, “surgical vascular creation”, “interventional vascular access creation” (questions 12, 16 and 17), response rates were inconsistently lower, shown as additional light-coloured bars.

**Table S1: Test for influencing factors of satisfaction**

| **Influencing factor** | **AV fistulas** | **tHDC** | **PD** |
| --- | --- | --- | --- |
| Structured cooperation | 0.06 | 0.74 | 0.13 |
| Radiology with expertise | 0.91 | 0.49 | 0.07 |
| Vascular surgery with expertise | 0.07 | 0.18 | 0.22 |
| Certification | 0.12 | 0.52 | 0.41 |
| Hospital level | 0.31 | 0.62 | 0.36 |

An univariate proportional odds models was used to investigate the association of possible influencing variables (availability of vascular surgery or radiology, certification, structured cooperation) on satisfaction. For this purpose, satisfaction was categorized into three levels (very good–good, sufficient, poor–inadequate). The p-values of the overall test of each variable are listed by category. None of the factors showed a significant association.

AVF: arterio-venous fistulas, tHDC: tunnelled haemodialysis catheters, PD: peritoneal dialysis catheters
